# Mother-daughter dyadic approach for starting preconception counseling at puberty in girls with diabetes

**DOI:** 10.7243/2054-9865-1-2

**Published:** 2014-12-29

**Authors:** Denise Charron-Prochownik, Andrea Rodgers Fischl, Jessica Choi, Patricia L. Schmitt, Neil H. White, Dorothy Becker, Julie Downs, Margaret Hannan, Jennifer Thurheimer, Susan M. Sereika

**Affiliations:** 1 Department of Health Promotion & Development, University of Pittsburgh, School of Nursing, USA; 2 Department of Pediatrics, Washington University in St. Louis, and St. Louis Children's Hospital, St. Louis, Missouri, USA; 3 Department of Pediatrics, Children's Hospital of Pittsburgh of UPMC, University of Pittsburgh, USA; 4 Center for Risk Perception and Communication, Carnegie Mellon University, USA; 5 Center for Research and Evaluation, School of Nursing, University of Pittsburgh, USA

**Keywords:** Diabetes mellitus, reproductive health, adolescence, mother-child relation, family planning

## Abstract

**Background:**

Preconception counseling (PC) significantly and inexpensively reduces risks of reproductive-health complications for women with diabetes. Our validated technology-based preconception counseling intervention, *READY-Girls*, is tailored for female teens with type 1 (T1D) and type 2 (T2D) diabetes and targets decision-making regarding effective family planning and seeking PC. Our teen-focused research was instrumental in changing the American Diabetes Association's Practice Recommendations to specify that preconception counseling should “Start at puberty…”. This directive requires support from well-informed mothers of teens. Our goal is to provide both teen girls and their mothers with preconception counseling knowledge, and provide mothers with sex-communication training. Evaluation should focus on mother-daughter dyads.

**Purpose:**

This feasibility study explored mother's and daughter's awareness and knowledge of diabetes and pregnancy, and preconception counseling; and compared mother-daughter responses using dyadic analyses.

**Methods:**

A mixed-method design was conducted with 10 mothers of daughters with T1D. Mothers were given *READY-Girls* intervention and completed knowledge and support questionnaires. Their responses were compared to those of their daughter's who were participating in a large randomized, control intervention trial with *READY-Girls*.

**Results:**

The major theme from one-on-one interviews was, “I know nothing about diabetes/pregnancy risks and PC”. Mother's and daughter's perceptions of having limited knowledge were confirmed by low knowledge scores. Mothers perceived giving higher levels of support compared to their daughter's perceptions of receiving support.

**Conclusion:**

Mothers can play a vital role in initiating discussions regarding reproductive-health with their daughters and reinforcing preconception counseling. Mother-daughter team approach for starting preconception counseling at puberty in girls with diabetes is feasible. Mother-daughter dyadic analyses can be important to explore possible mediating and moderating roles of mother-daughter communication and support about reproductive health on the relationship between *READY-Girls* intervention and sustainable outcomes.

## Background

Women with diabetes and their off spring are at risk of perinatal complications due to uncontrolled blood sugars [1]. Preconception counseling (PC) can significantly and inexpensively reduce risks of reproductive-health complications in women with diabetes by providing information and skills to plan a pregnancy when it's safe and wanted, and help women achieve euglycemia before and during a pregnancy [1].

Our technology-based PC intervention called *READY-Girls* (Reproductive-health Education and Awareness of Diabetes in Youth-Girls) is a validated PC program, available in DVD and book formats [2], based on the Expanded Health Belief Model [4], and developed for female adolescents with diabetes. *READY-Girls* is tailored for female teens with type 1 (T1D) and type 2 (T2D) diabetes and targets decision-making regarding effective family planning and seeking PC [2]. Our teen-focused research was instrumental in changing the American Diabetes Association's (ADA) Practice Recommendations to specify that PC should “Start at puberty…” [1,3]. This directive requires support from well-informed mothers of teens. Parent-adolescent communication has been associated with positive sexual health outcomes among teenage girls, namely, delaying sexual initiation and decreasing teen pregnancies [5]. Because mothers have a critical role in providing reproductive health information [5], our goal is to provide both teen girls with diabetes and their mothers with preconception counseling and knowledge, and provide mothers with sex-communication training. Evaluation should focus on mother-daughter dyads. Therefore, the purpose of this feasibility study was to explore awareness and knowledge of diabetes and pregnancy, and PC in mothers and daughters with diabetes; mother's support; and compare mother-daughter responses using dyadic analyses.

## Methods

*READY-Girls* was tested in our original study, an independent randomized controlled trial (RCT) from 2 sites by 113 adolescent females with T1D between the ages of 13 to 20 years. Details and results are described elsewhere [6]. A mixed-methods design was used in this feasibility sub-study with 10 randomly chosen biological mothers of daughters with diabetes from the *READY-Girls* Intervention Study. At the conclusion of the intervention trial, mothers were interviewed by the project director using the following 3 open-ended items: *What do you know about diabetes and pregnancy? What do you know about diabetes and birth control? What do you know about preconception counseling and care?*

Following the interview, the mothers were given the *READY-Girls* book intervention. Close-ended measures of knowledge and social support were completed using paper and pencil questionnaires. Baseline data of the daughter's responses from parallel questionnaires from the *READY-Girls* Intervention Study were compared to those of their mothers.

Knowledge was assessed using a 76 item multiple choice test, based on 100% correctness [7]. It included the following subscales: diabetes and pregnancy (28 items); contraception (5 items); sexuality (7 items); puberty (3 items); PC (25 items); and general family planning (8 items). The internal consistency using Cronbach alpha was 0.71 and test-retest reliability r=0.76. Split-half differentiates pre- from post-test [7].

Social Support was measured by the Social Support scale from the *Reproductive Health and Diabetes Questionnaire* Social support is the process by which help is obtained from the social network (e.g., mothers/female guardian) to meet one's needs [8]. Support measure for mothers is the perceived actual support (emotional, appraisal, informational, and instrumental) [8] they provided to their daughters for lifestyle management and family planning vigilance. Daughter's measure is perceived available support from their mother for the same behaviors. Items have Likert-type scaling with response choices of “a lot of help”=7 to “no help at all”=1. Scores are summated (range 6-42) where higher scores suggest greater support. Internal consistency is high with a Cronbach's alpha of 0.92 [9].

The three questions from open-ended items were qualitatively analyzed. Categories were derived from content analysis. Two members of the research team reviewed and rated the responses until mutual agreement was achieved. Summation scores from knowledge and social support measures were quantitatively analyzed. Descriptive and comparative statistics were used to examine differences between mother and daughter knowledge and social support scores within the mother-daughter dyad using either paired-t test or Wilcoxon signed-rank test with exact estimation of p-values. The level of significance was set at 0.05.

Consents/assents were obtained from both mothers and daughters. Both the adolescent RCT and the mother's mix-method sub-study were approved by the institutional review boards.

## Results

Mother and daughter demographic characteristics are reported in Table 1. The majority was Caucasian; and the majority of mothers was married and had at least some college education. One mother developed T2D after the age of 40 years, and another mother had gestational diabetes.

Qualitative themes from one-on-one interviews are presented in Table 2. The most frequent response from both mothers and daughters regarding their understanding of these topics was, “*Nothing*”. For example, about a fifth of the mothers and daughters knew nothing about *diabetes and pregnancy.* And approximately half of the mothers and daughters responded that they knew, “*Nothing*” or “*Not much*” to the question, *What do you know about diabetes and birth control?* The mother with T2D reported not knowing this information; while the mother with gestational diabetes was also a nurse, and was aware of the complications and effects of diabetes on pregnancy and birth control. All mothers stated that the *READY-Girls* program was important for their daughters.

Mother's and daughter's perceptions of having limited knowledge was confirmed by low knowledge scores (<80% correct). Similar low to moderate levels of knowledge were observed between mothers and their daughters. Although not significant, a trend was noted (p=0.076). With regards to perceived social support, mothers reported providing high levels of support to their daughters; in contrast, daughter's perceived receiving lower levels of support that were more variable from dyad to dyad (p=0.002). See Table 3 for mother-daughter within dyad differences.

## Discussion and conclusion

A mother-daughter team approach for starting PC at puberty in girls with diabetes is feasible. Dyadic differences and similarities in mother-daughter responses were noted in our study. Although mother's overall knowledge scores tended to be slightly higher than their daughters, both averages were low. Conversely, support scores were significantly different. Family-based interventions to promote healthy practices have used mother-daughter dyads. Arrendondo et al., [10] taught mothers to support their daughters behavioral change efforts to promote physical activity. Other researchers have utilized the mother-daughter dyad social support system to show efficaciousness for interventions related to diet, lifestyle, substance use and sexual topics [11-13].

This study was a feasibility study with a mixed-method design. As a feasibility study it had limitations. Although the sample was adequate for qualitative analyses, larger samples are needed for quantitative analyses. The sample was recruited from 2 sites, therefore, limiting the generalizability. The sample only included teens with T1D. However, women with T2D and gestational diabetes are also at risk of the same perinatal complications, and therefore, could benefit from receiving preconception counseling and achieving euglycemia before and during a pregnancy [1]. Despite some limitations, this study had several strengths. This study collected both qualitative and quantitative data to confirm and enrich the findings. It was innovative to combine data from both mothers and daughters on this significant topic by applying dyadic analyses methods. Dyadic analysis focuses on the non-independence between and within dyads, pairs of individuals that are related and distinguishable like mother and child [14]. When the unit of analysis is the dyad, the natural dependencies between both members of the dyad are taken into account [14]. This is particularly true when conducting research in pediatric and adolescent diabetes populations; where parents can influence health behavior and outcomes in youth with diabetes [14].

Our future goal is to provide both diabetic teen girls and their mothers with preconception counseling and knowledge, and provide mothers with sex-communication training. Mothers can play a vital role discussing reproductive-health with their daughters and reinforcing PC [6,15]. Mother-daughter dyadic analyses can be important to explore possible mediating and moderating roles of mother-daughter communication and support about reproductive health on the relationship between *READY-Girls* intervention and sustainable outcomes. This research could set new standards of practice for self-management education of adolescent females with diabetes [6].

## Figures and Tables

**Table 1:** Mother-daughter (M-D) sample characteristics

Characteristic	Mother Mean±SD/n (%)	Daughter Mean±SD/n (%)
Age (years)	48.4±2.8	15.3±1.1
Race: White, n (%)	9 (90) a	10 (100)
Diabetes status, n (%)	2 (20)	10 (100)
Education: At least some college, n (%)	6 (60)	0
Marital Status: Husband, n (%)	7 (70)	0
Income: <$20,000/year, n (%)	1 (10)	N/A
Religion: Roman Catholic, n (%)	4 (40)	5 (50)

a One mother did not provide a response to race

**Table 2:** Qualitative results: M-D open-ended responses

Question #1: What do you know about diabetes and pregnancy?		

Answers	Mother	Daughter
Nothing/Not much/Very little	19%	24%
Blood sugar should be well controlled	25%	24%
Dangerous/complicate/Risk for both mother and child	25%	6%

**Question #2: What do you know about diabetes and birth control?**		

**Answers**	**Mother**	**Daughter**
Nothing, Not much	54%	58%
BC has its own side effects (e.g., high BP and blood clots)	15%	0%
Important to take BC pill	0%	18%

**Question #3: What do you know about preconception counseling and care?**		

**Answers**	**Mother**	**Daughter**
Nothing, Not much, never heard of it	38%	88%
Very important	23%	0%
Necessary due to possible complications	15%	0%

**Table 3:** Quantitative results: within-dyad diferences (N=9)

Variables	Mother	Daughter	Diference	p
Total knowledge (% correct)	71.6±4.86^a^	67.0±5.9	4.6±6.7	0.076^c^
	67.4-81.4	55.8-74.4	4.7-14.0	--
Perceived social support (Sum)	41.8±0.5	31.7±7.2	10.2±6.8	0.002^c^
	42.0^b^	33.0	9.0	0.008^d^
	40.5-42.0	17.0-42.0	0-23.5	--

a Mean±SD and range of responses reported as minimum–maximum;

b Median;

c Based on paired-t test;

d Based on Wilcoxon signed-rank test with exact estimation of p-values

## References

[1] American Diabetes Association (2014). Standards of medical care in diabetes-2014. Diabetes Care.

[2] Charron-Prochownik D, Downs J (2014). Diabetes and Reproductive Health for Girls.

[3] (2009). Standards of medical care in diabetes--2009. Diabetes Care.

[4] Burns AC (1992). The expanded health belief model as a basis for enlightened preventive health care practice and research. J Health Care Mark.

[5] Barnes H, Olson D (1985). H. Parent-Adolescent Communication and the Circumplex Model. Child Development.

[6] Charron-Prochownik D, Sereika SM, Becker D, White NH, Schmitt P, Powell AB, Diaz AM, Jones J, Herman WH, Fischl AF, McEwen L, DiNardo M, Guo F, Downs J (2013). Long-term effects of the booster-enhanced READY-Girls preconception counseling program on intentions and behaviors for family planning in teens with diabetes. Diabetes Care.

[7] Downs J, Bruine de Bruin W, Moltz K, Charron-Prochownik D (2008). Adolescents' Knowledge Associated with Metabolic Control and Intentions to Seek Preconception Counseling. Diabetes.

[8] Tardy C (1985). Social support measurements. American Journal of Community Psychology.

[9] Charron-Prochownik D, Wang SL, Sereika SM, Kim Y, Janz NK (2006). A theory-based reproductive health and diabetes instrument. Am J Health Behav.

[10] Arredondo EM, Morello M, Holub C, Haughton J (2014). Feasibility and preliminary findings of a church-based mother-daughter pilot study promoting physical activity among young Latinas. Fam Community Health.

[11] Sang J, Cederbaum JA, Hurlburt MS (2014). Parentification, substance use, and sex among adolescent daughters from ethnic minority families: the moderating role of monitoring. Fam Process.

[12] Schwinn TM, Schinke S, Fang L, Kandasamy S (2014). A web-based, health promotion program for adolescent girls and their mothers who reside in public housing. Addict Behav.

[13] Sorkin DH, Mavandadi S, Rook KS, Biegler KA, Kilgore D, Dow E, Ngo-Metzger Q (2014). Dyadic collaboration in shared health behavior change: the effects of a randomized trial to test a lifestyle intervention for high-risk Latinas. Health Psychol.

[14] Kenny DA, Kashy DA, Cook WL (2006). Dyadic Data Analysis.

[15] Hannan M, Happ MB, Charron-Prochownik D (2009). Mothers' perspectives about reproductive health discussions with adolescent daughters with diabetes. Diabetes Educ.

